# Assessing focal spot alignment in clinical linear accelerators: a comprehensive evaluation with triplet phantoms

**DOI:** 10.1007/s13246-024-01450-9

**Published:** 2024-07-02

**Authors:** Hans L. Riis, Kenni H. Engstrøm, Luke Slama, Joshua Dass, Martin A. Ebert, Pejman Rowshanfarzad

**Affiliations:** 1 Department of Oncology, Odense University Hospital, Odense, Denmark; 2 Department of Clinical Research, University of Southern Denmark, Odense, Denmark; 3 Department of Radiation Oncology, Sir Charles Gairdner Hospital, Nedlands, WA 6009 Australia; 4 Centre for Advanced Technologies in Cancer Research (CATCR), Perth, WA 6000 Australia; 5 School of Physics, Mathematics, and Computing, The University of Western Australia, Crawley, WA 6009 Australia; 6 Radiofysisk Laboratorium, Odense University Hospital, Kløvervænget 19, DK-5000 Odense C Odense, Denmark

**Keywords:** Focal spot, MV radiation, Long-term stability, Linear accelerator, Gantry rotation, EPID, STARCHECK, arcCHECK

## Abstract

A fundamental parameter to evaluate the beam delivery precision and stability on a clinical linear accelerator (linac) is the focal spot position (FSP) measured relative to the collimator axis of the radiation head. The aims of this work were to evaluate comprehensive data on FSP acquired on linacs in clinical use and to establish the ability of alternative phantoms to detect effects on patient plan delivery related to FSP. FSP measurements were conducted using a rigid phantom holding two ball-bearings at two different distances from the radiation source. Images of these ball-bearings were acquired using the electronic portal imaging device (EPID) integrated with each linac. Machine QA was assessed using a radiation head-mounted PTW STARCHECK phantom. Patient plan QA was investigated using the SNC ArcCHECK phantom positioned on the treatment couch, irradiated with VMAT plans across a complete 360° gantry rotation and three X-ray energies. This study covered eight Elekta linacs, including those with 6 MV, 18 MV, and 6 MV flattening-filter-free (FFF) beams. The largest range in the FSP was found for 6 MV FFF. The FSP of one linac, retrofitted with 6 MV FFF, displayed substantial differences in FSP compared to 6 MV FFF beams on other linacs, which all had FSP ranges less than 0.50 mm and 0.25 mm in the lateral and longitudinal directions, respectively. The PTW STARCHECK phantom proved effective in characterising the FSP, while the SNC ArcCHECK measurements could not discern FSP-related features. Minor variations in FSP may be attributed to adjustments in linac parameters, component replacements necessary for beam delivery, and the wear and tear of various linac components, including the magnetron and gun filament. Consideration should be given to the ability of any particular phantom to detect a subsequent impact on the accuracy of patient plan delivery.

## Introduction

To meet the needs for accurate, high-quality radiotherapy treatments including stereotactic approaches [1], quality assurance (QA) recommendations have been defined and continuously updated for linear accelerators (linacs) [1, 2]. QA requirements have to accommodate the variety of beam delivery approaches, spanning uniform-intensity through to intensity-modulated radiotherapy, volumetric modulated arc therapy [2] (VMAT) and flattening-filter-free (FFF) beam [3] deliveries at high dose rates. Built-in electronic-portal-imaging-devices (EPIDs), which enable MV image acquisition, serve as valuable tools for linac QA. Furthermore, the combination of EPID with phantoms or other external detection systems is widely employed for QA purposes [4–7].

Recommended linac QA procedures [1] do not specifically define tolerances for the focal spot position (FSP) in relation to the axis of the beam limiting device (BLD), such as a collimator, which is a fundamental parameter for precise beam deliveries. The focal spot (FS) serves as the source of X-ray generation (bremsstrahlung) when high-energy electrons decelerate within the high Z-material target. The size of the FS is around two millimetres in diameter [8]. The FSP is ideally on the collimator rotation axis regardless of the gantry angle. Regular QA assessments of the FSP are uncommon, with the primary focus being on beam symmetry, flatness, field size, and radiation isocentre location. These aspects are under consideration in linac QA procedures as an indirect approach to FSP QA; however, a need to assess FSP directly and identify action levels related to FSP has been established [9, 10].

The FSP will have an impact on the calibration of MLCs and jaws and the radiation isocentre position. When the bremsstrahlung photon beam is not centred relative to the symmetry axis of the flattening filter, it can cause a tilting effect on the X-ray radiation field profile. This tilt can be compensated by adjusting the angle at which the electron beam strikes the target or by repositioning the FSP on the target. Even for the FFF energies, it may be necessary to adjust the electron angle to achieve symmetric beams. Therefore, it is important to compensate for filter misalignments and other imperfections by ensuring that the electron current strikes the high-Z target material at a non-oblique angle, ensuring a symmetric beam and maintaining the FSP on the collimator axis [11]. Ideally, the FSP should always be located on the rotational axis of the collimator at all gantry angles.

In treatment planning systems (TPSs), it is commonly assumed that the central axis of all beam-limiting devices aligns with the collimator axis at all gantry angles. This assumption implies that there is no sag in the beam limiting device and that the FSP remains positioned on the collimator axis for every gantry angle.

Monitoring the FSP without the use of a phantom can be achieved by analysing the positions of the jaws and MLC at gantry angle 0°, [12, 13]. However, it should be noted that the beam-limiting devices may be influenced by gravity during gantry rotation, making them unreliable for obtaining absolute FSP measurements at other gantry angles. To address this issue, various studies have explored the assessment of the FSP using phantoms placed on the treatment couch [14] or attached to the gantry head [15–17] of the linear accelerator. To measure the FSP in relation to the collimator axis, it is necessary to detect the position of the collimator axis. This can be obtained by imaging a rigid phantom that is affixed to the radiation head during collimator rotations [16, 17].

This work aimed to analyse comprehensive data acquired from linacs in clinical use to assess the long-term stability and precision of the FSP relative to the collimator axis. The findings from this analysis would provide valuable insights into the necessity of routine FSP assessment and aid in establishing appropriate assessment frequencies and tolerances. Furthermore, the sensitivity of the FSP to variations in dose rate and the direction of gantry rotation was investigated. In addition, two commercially available phantoms were included in the study to validate their ability to detect subsequent impact on treatment plan delivery.

## Materials and methods

### Linacs

A summary of the linacs used is given in Table 1. FSP measurements were conducted on a total of eight Philips/Elekta (Elekta AB, Stockholm, Sweden) linacs, comprising five linacs with 6 MV and 18 MV energies, and three linacs with 6 MV, 18 MV and 6 MV FFF energies. The linacs equipped with three energies were all fitted with the Agility gantry head (MLC160), while the other linacs were equipped with the MLCi or MLCi2 heads. All the linacs were equipped with Perkin Elmer (PerkinElmer, Waltham, MA) amorphous silicon (a-Si) detectors, featuring a sensitive area of 41 × 41 cm^2^ and a resolution of 1024 × 1024 pixels. All the linacs in this study were equipped with PerkinElmer XRD 1640 AL panels. However, the AL panels on FFF linacs were replaced during this work with the high dose rate models, PerkinElmer XRD 1642 AP, which support the acquisition of images of FFF beams at the nominal dose rate. The sensitive area and the number of pixels of the two types of panels are the same. The distances between the exit side of the high-Z target material and the upper and lower edges of the different beam-limiting devices are displayed in Table 2. These distances are valuable for evaluating the impact of the radiation head model on the FSP offset relative to the collimator axis.

**Table 1:** Overview of the equipment: linac models, MLC models, energies and EPID panel models. Some AL EPID panels were replaced with the AP panel in 2017. These are marked with AL/AP in the table. Each linac is referred to by the labels A-H

Linac label	Linac model	MLC model	Energies (MV)	EPID panel model
A	Elekta Synergy	MLCi	6, 18	AL
B	Philips SL 20	MLCi	6, 18	AL
C	Elekta Synergy	MLC160	6, 18, 6 FFF	AL/AP
D	Philips SL 20	MLCi	6, 18	AL
E	Elekta Synergy	MLCi2	6, 18	AL
F	Elekta Synergy	MLCi2	6, 18	AL
G	Elekta Versa HD	MLC160	6, 18, 6 FFF	AL/AP
H	Elekta Versa HD	MLC160	6, 18, 6 FFF	AL/AP

**Table 2:** Distances measured from the exit side of the high-Z target material to different edges of beam limiting devices (BLDs) for MLCs and jaws in selected Elekta linac radiation heads. See the acknowledgement section for reference

Radiation head	MLCi (mm)	MLCi2 (mm)	MLC160 (mm)
MLC (Upper edge)	298.0	293.0	311.8
MLC (Lower edge)	373.0	375.0	401.8
${{\ell}}_{\text{MLC}}$ (MLC midpoint)	335.5	334.0	356.8
Jaw (Upper edge)	431.0	431.0	432.0
Jaw (Lower edge)	509.0	509.0	509.0
${{\ell}}_{\text{Jaw}}$(Jaw midpoint)	470.0	470.0	470.5

The servo in the *x*-direction was disabled on all our linacs at the time of measurements with the rigid phantom. It is recommended by the Elekta company to enable the lateral (*x*-component) beam servo for the energies 6 MV and 18 MV and disabling it for 6 MV FFF. The enabling was carried out shortly after the latest measurements with the rigid phantom for 6 and 18 MV. When the *x*-component is not regulated via a servo, the beam steering relies solely on a set value, 2 T I ctrl, at the actual gantry angle. Corrections to the set values, as the gantry rotates, are based on lookup tables. Inaccurate lookup tables can result in an unintended offset of the FSP during gantry rotation. Furthermore, the calibration of the lookup table is acquired at one dose rate level, typically at the highest dose rate. The calibration of the lookup table is carried out in the linac learning mode to read out the 2 T I ctrl and 2R I ctrl values at low gantry speed. While 6 MV and 18 MV have their own lookup table, 6 MV FFF uses the lookup table of 6 MV. This feature may result in different locations of the FS for 6 MV and 6 MV FFF beams.

Our room lasers are adjusted to the isocentre of 6 MV, which might have a slightly different shift compared to 6 MV FFF. The isocentre location of the 6 MV beam was determined using eight beams at the four cardinal gantry angles, each at both collimator $-$ 90° and 90° [18, 19].

The imaging acquisition using the AL and AP panels was compared. The comparison was carried out by counting the number of usable frames registered by the image acquisition software iViewGT. During image acquisition, a running count for the total number of frames was shown in the lower left corner of the iViewGT monitor. This comparison was conducted on all the investigated linacs, and the number of frames was counted as a function of monitor units and the set dose rate. The frame counting was carried out on the latest version of the EPID panel of each linac.

The gantry, collimator angle, and coordinate axis definitions used to characterise the linac followed IEC standard [20].

### Ball-bearing phantom

**Fig. 1:**
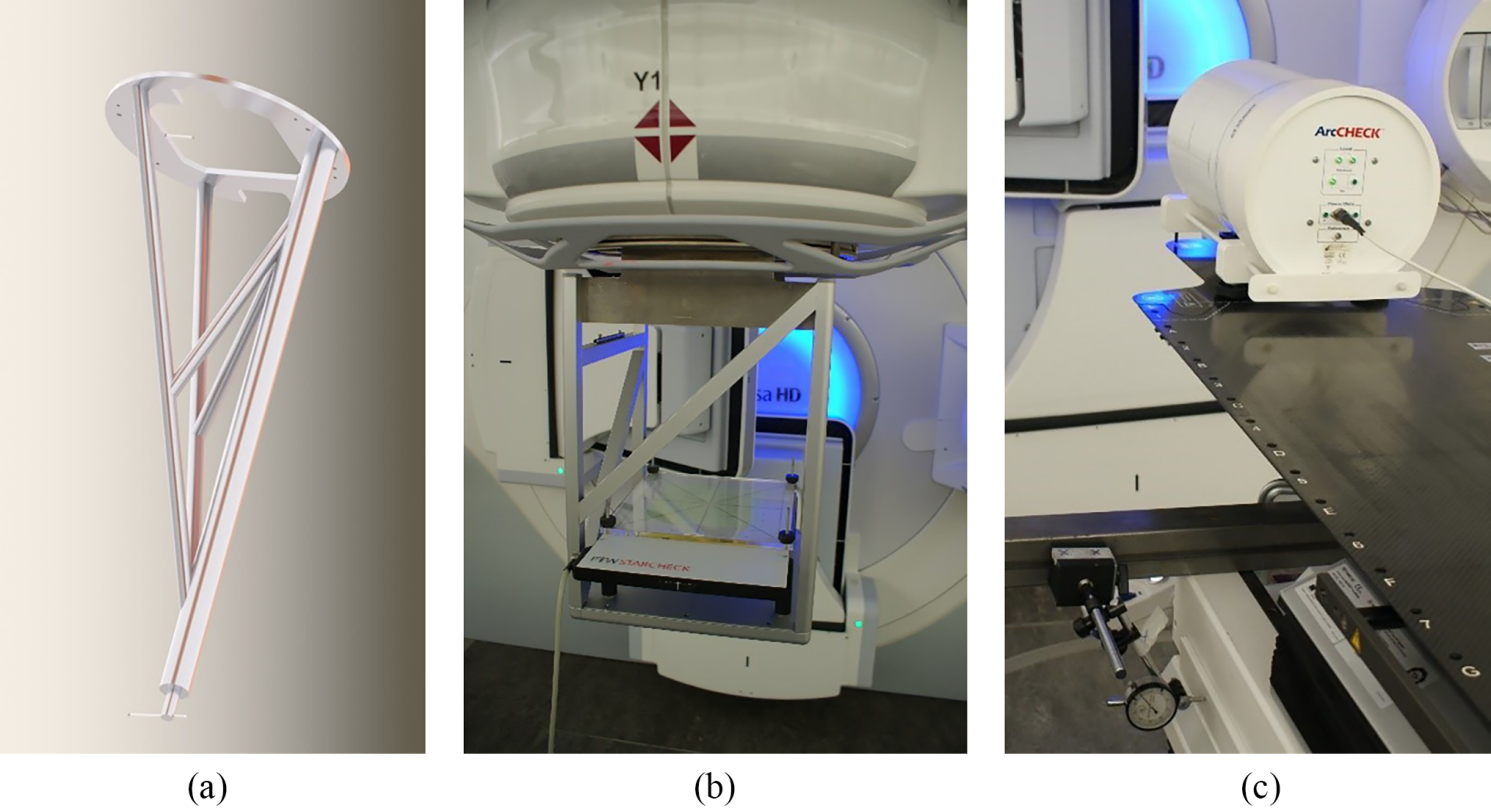
The three phantoms that have been applied in this work. (**a)** The specially designed phantom in 3D view rendered from SOLIDWORKS (SolidWorks Corporation, Waltham, MA) drawings of the phantom. The phantom supports two ball-bearings, one near the attachment plate and one at the end of the rod (opposite the attachment plate). The two ball-bearings and the attachment plate for mounting on the radiation head of an Elekta linac can be seen. (**b)** The PTW STARCHECK phantom is attached to the radiation head of an Elekta Versa HD linac. An extra build-up of 1 cm PMMA is placed on top of the phantom. (**c)** ArcCHECK is positioned at the isocentre of an Elekta Versa HD, linac G. One of three dial indicators measuring the lateral shift of the couch is shown. The indicator is held by a magnetic base attached to a steel rod

Figure 1a depicts the ball-bearing phantom used for detecting the FS, which consists of two tungsten carbide ball-bearings positioned at two different distances. A detailed description of the design of the design of this ball-bearing phantom can be found elsewhere [16]. The linac gantries were systematically rotated either clockwise (CW) or counter-clockwise (CCW) through a complete 360° in steps of 30°. At each gantry angle, the collimator was rotated 360° CW or CCW in steps of 30°. At each combination of gantry angle, $\varphi$, and collimator angle, $\theta$, an EPID image was acquired. In each series, a total of 169 images were captured, corresponding to each position ($\varphi$, $\theta$) where the ball-bearings were exposed. The beams were delivered at the nominal dose rate, except for the 6 MV FFF exposures to the AL panel, which were delivered at 25% of the nominal dose rate to avoid EPID saturation. For the AP panel, the dose rate remained at the nominal value. On Agility linacs, images were acquired for both CW and CCW rotations of the gantry and collimator. Measurements were conducted to investigate the influence of the number of MUs on the imaging and subsequent analysis on one linac with 3, 10 and 50 MUs per segment. In all other measurements, 10 MUs were used in each segment.

The 169 beams were delivered in service mode using a step-and-shoot technique, controlled by the Linac Control System (LCS) Integrity (Elekta AB, Stockholm, Sweden). Integrity allows a maximum of 254 control points, which correspond to 127 beams. Therefore, the 169 beams were delivered in two step-and-shoot series: 127 and 42 in the CCW gantry rotation and 42 and 127 in the CW direction. During CCW rotation, the beam started at $\left(\varphi ,\theta \right)=\left(180^\circ ,180^\circ \right)$ and ended at $\left(\varphi ,\theta \right)=\left(-90^\circ ,-90^\circ \right)$ after 127 beams. The next series started at $\left(\varphi ,\theta \right)=\left(-90^\circ ,-120^\circ \right)$ and ended at $\left(\varphi ,\theta \right)=\left(-180^\circ ,-180^\circ \right)$ after 42 beams. In CW rotation, the beam started at $\left(\varphi ,\theta \right)=\left(-180^\circ ,-180^\circ \right)$ and ended at $\left(\varphi ,\theta \right)=\left(-90^\circ ,-120^\circ \right)$ after 42 beams. The next series started at $\left(\varphi ,\theta \right)=\left(-90^\circ ,-90^\circ \right)$ and ended at $\left(\varphi ,\theta \right)=\left(180^\circ ,180^\circ \right)$ after 127 beams.

The images were analysed, and the FSPs were calculated using MATLAB code developed in-house (MathWorks, Natick, MA, USA) [16, 17].

### Phantom for quality control and linac beam adjustment

The PTW STARCHECK T10043 array (PTW, Freiburg, Germany) was used to measure the radiation field centre (RFC) [21] and beam symmetry during gantry rotation [22]. The array was mounted on a rigid rack, which was custom-made and attached to the radiation head of the linac (see Fig. 1b). The detector array weighed 5.5 kg, and with the rack and an additional 1.0 cm thick polymethyl methacrylate (PMMA) build-up plate, the total weight was 13.3 kg.

Data were collected during gantry rotation using the PTW software module BeamAdjust [22]. The data acquisition was carried out in the ‘‘Movie’’ mode of BeamAdjust with the average-off setting for dose rate acquisition. The beam was delivered in service mode on the linac with interlocks turned off at a nominal dose rate and a maximum gantry rotation speed of approximately 6°/s. Each acquisition lasted 0.4 s, equivalent to a gantry rotation of 2.4° with 150 profiles read out during a complete 360° gantry rotation. The sensitive area of the array was positioned at the source-detector-distance (SDD) of 100 cm, with a build-up of 1.85 cm (1.0 cm PMMA plate and 0.85 cm intrinsic). The sensitive area consists of 527 vented ionisation chambers with a volume of 0.05 cm^3^. The field size was 20 × 20 cm^2^. The number of detectors in the cross-plane (CR) and in-plane (IN) directions was 83 each.

The PTW STARCHECK phantom was attached to the radiation head of the linac. The phantom was aligned with the cross-hair indicating the collimator rotation axis and the orientation of the BLDs. The collimator angle was set to 0°. The detector array acquisition was configured in dose rate MOVIE mode [23]. The linac was set to the time (ctrl T) interlock feature within the Integrity software. A 20 × 20 cm^2^ field was delivered at a nominal dose rate while the gantry rotated at a constant speed. The measurements and gantry rotation of the linac were initiated and maintained manually, resulting in minor uncertainties in the synchronisation of the gantry angle.

The linac gantry was turned to the start position at a gantry angle 180°. The ending gantry angle − 180° was manually entered into the LCS Integrity software. The radiation beam was turned on, and the measurement was initiated by selecting ‘‘start’’ in the BeamAdjust software. Shortly after the activation of data acquisition, the gantry rotation was started by pressing the return key on the LCS keyboard while simultaneously pressing the gantry and collimator rotation key and the dead man key on the linac function keypad. Once the gantry reached − 180°, the measurement was stopped by pressing ‘‘stop’’ in the BeamAdjust software.

The BeamAdjust software does not have the capability to extract gantry angles; however, it registers the time for each readout. Furthermore, BeamAdjust does not include a plotting function. Therefore, the ‘‘Movie’’ file (PTW Mephysto*.mcc file format) [24] was analysed in MATLAB (MathWorks, Natick, MA, USA). The radiation RFC and beam symmetry were calculated as a function of time. With a constant gantry angle rotation speed, the time was transferred to the gantry angle and plotted accordingly. The RFC was determined relative to the centre of the STARCHECK phantom, considering both IN and CR components. In this context, IN ($y$) and CR ($x$) refer to an ($xy$)-coordinate system that rotates with the gantry but aligns with the fixed *XY*-room coordinate system [20] at $\varphi =0^\circ$ (see subsection 'Phantom for treatment plan quality measurement'). Furthermore, the beam symmetry was calculated using the area ratio method [22], relative to the measured RFC as a function of the gantry angle. It is important to note that the symmetry definition used in this study is precisely twice as high as the definition used elsewhere [25].

### Phantom for treatment plan quality measurement

The ArcCHECK phantom, Model 1220 (FFF compatible) from Sun Nuclear Corporation (SNC) (Melbourne, FL), was utilised to provide a clinical perspective on the FS alignment results. The phantom features 1386 precision diode detectors (size 0.8 × 0.8 × 0.03 mm^3^) arranged in a helical formation on a cylinder with a radius of 7.6 cm and a length of 21 cm. The detectors are situated in PMMA material at a physical depth of 2.9 cm from the outer surface of the phantom, positioned at the corners of squares of 1 × 1 cm^2^. The SNC Patient Software Version 8.3 was employed for the subsequent analyses [26, 27]. Out of the detectors present in our ArcCHECK phantom, two detectors were found to be non-functional and therefore excluded analyses. The phantom was set up using the head and neck extension (iBEAM® evo Extension H&N) for the iBEAM® evo Couchtop EP (Medical Intelligence, Schwabmünchen, Germany). After aligning the room lasers to point at the radiation isocentre of the linac, the ArcCHECK phantom was positioned according to the reference position marked on the phantom, see Fig. 1c. The levelling of the phantom was checked using a spirit level. To measure the shifts in the $X$, $Y$ and $Z$ directions of the ArcCHECK phantom, three Mitutoyo dial indicators (Kanagawa, Japan) were attached to the couch using a magnetic socket. The dial indicators used for measurement had a readout precision of 0.01 mm. Small shifts were incrementally applied in sub-millimetre steps ($<$ 1 mm) in each direction, while the other two coordinates were kept fixed at the isocentre. The maximum total shifts applied were $\pm 2$ mm, which is significantly smaller than the spacing within the detecting grid. These shifts are defined within a right-handed room ($XYZ$)-coordinate system [20] with $Y$ representing the positive direction towards the gun and $Z$ representing the positive direction upwards.

Three VMAT treatment plans were administered over a complete arc of $360^\circ$ gantry rotation—a 6 MV plan specifically designed for a head and neck case; a 6 MV FFF plan intended for a stereotactic pancreas case; and an 18 MV plan utilised for QA purposes. It is important to note that all the plans were delivered with the collimator angle set at 15°.

In the SNC software, the lower-dose threshold ($\rm LDT$) was configured to 10%. This setting is consistently used for ArcCHECK testing of all clinical plans in our department. The purpose of setting the threshold at this level is to eliminate the influence of scattered radiation and noise arising in measured data. The measurements were compared to the reference setup of the phantom, which implies that the alignment lines on the phantom coincided with the room laser lines at the position $(X, Y, Z)=(\text{0,0},0)$ mm, which corresponds to the radiation isocentre of the linac. The ArcCHECK measurements were performed on one linac only.

### Theory

The position of the FS, denoted as $\mathbf{d}=\left({d}_{x}\left(\varphi \right), {d}_{y}\left(\varphi \right)\right)$, with respect to the rotational axis of the collimator, ${\mathbf{C}}_{0}$, can be measured using BLD devices within the radiation head. These devices include jaws and MLCs located at varying distances from the FS [12, 13].

The relationship between the FS offset, $\mathbf{d}$, and the gap between the central axis of the BLD and the collimator axis at an isocentre distance, ${\mathbf{C}}_{\text{BLD}}-{\mathbf{C}}_{0}$, can be obtained through geometrical considerations. [16]

1 $$\mathbf{d}= -\frac{{{\ell}}_{\text{BLD}}}{{{\ell}}_{\text{I}}-{{\ell}}_{\text{BLD}}}\left({\mathbf{C}}_{\text{BLD}}-{\mathbf{C}}_{0}\right)$$

with ${{\ell}}_{\text{I}}$ representing the isocentre (I) distance from the FS, and ${{\ell}}_{\text{BLD}}$ the BLD distance from FS. The central axis position (CAP) at the isocentre distance defined for the BLD jaws and MLCs is labelled ${\mathbf{C}}_{\text{BLD}}$. The parameters $\mathbf{d}$, ${\mathbf{C}}_{0}$, ${\mathbf{C}}_{\text{BLD}}$, Eq. (1), are given in the ($xy$)-plane of the ($xyz$)-coordinate system with the $z$-axis representing the rotational axis of the collimator ${\mathbf{C}}_{0}$, and the $y$-axis parallel to the $Y$-axis. The ${\mathbf{C}}_{\text{BLD}}$ represents the zero point for BLD calibrations, typically carried out at a gantry angle of 0°. An FSP misalignment $\left|\mathbf{d}\right|\ne 0$ results in an offset in the BLD calibration relative to the collimator axis ${\mathbf{C}}_{0}$.

The application of Eq. (1) uses the assumption that the BLD follows a circular pattern when rotated along with the radiation head of the linac. At least three collimator angles are needed to determine the location of ${\mathbf{C}}_{\text{BLD}}$. This has to be done for both the MLCs and the jaws, resulting in two equations with two unknowns derived from Eq. (1). Indeed, a mechanical sag of the jaws and MLCs can occur due to the influence of gravity as the gantry rotates [7, 28, 29]. As a result, the physical position of the BLD is expected to deviate from a perfectly circular pattern during gantry rotation. Nevertheless, it is assumed that the BLDs are not affected by gravity at gantry 0° and 180°, and their positions remain fixed during collimator rotations. Therefore, Eq. (1) is obeyed at the particular gantry angles of 0° and 180°.

The literature does not specify a particular tolerance level for $\left|{\mathbf{C}}_{\text{BLD}}-{\mathbf{C}}_{0}\right|$. However, findings from water tank measurements with ${\mathbf{C}}_{0}$ as a reference point, represented by the cross-wire of the light field, conclude that a tolerance level of $\left|{\mathbf{C}}_{\text{BLD}}-{\mathbf{C}}_{0}\right|\le$ 1 mm is reasonable. Thus, with a tolerance level of 1 mm, the position of ${\mathbf{C}}_{\text{BLD}}$ can be considered to lie within a circle of radius 1 mm centred at ${\mathbf{C}}_{0}$. The tolerance in the position of the FS in the target material, relative to the collimator axis, defines a circle of radius scaled by the factor ${{\ell}}_{\text{BLD}}/{({\ell}}_{\text{I}}-{{\ell}}_{\text{BLD}})$, according to Eq. (1).

The coincidence of the radiation and mechanical isocentre $\left|\Delta \mathbf{I}\right|$ is a parameter with tolerance levels defined in QA recommendations as baseline shifts relative to the value recorded during the commissioning of the linac [1]. Here, the $\Delta \mathbf{I}={\mathbf{I}}_{\text{R}}-{\mathbf{I}}_{\text{M}}=\left(\Delta {I}_{X},\Delta {I}_{Y},\Delta {I}_{Z}\right)$ denotes the position of the radiation isocentre ${\mathbf{I}}_{\text{R}}$ relative to the mechanical isocentre ${\mathbf{I}}_{\text{M}}$. The isocentre positions are determined in a fixed ($XYZ$)-room coordinate system with its origin at the mechanical isocentre ${\mathbf{I}}_{\text{M}}$, which is defined as $\left(\text{0,0},0\right)$ according to IEC 61217 [20].

It is assumed that the collimator axis intersects a longitudinal and horizontal line element through the mechanical isocentre in the $XYZ$ coordinate system during a 360° gantry rotation arising from gantry arm bending. Investigations on Elekta linacs have verified that the assumption was fulfilled within a lateral offset of less than 0.1 mm [30]. This line element can be described as $(0,{s}_{y} \text{cos}\varphi ,0)$ with $\left|{s}_{y}\right|=$ 0.6–0.8 mm for Elekta linacs [30] providing the average location of ${\mathbf{I}}_{\text{M}}$ over 360° at (0,0,0). The quantity $\Delta \mathbf{I}$ is defined by Eqs. (2))–(4) as.

2 $${\Delta \mathbf{I}= \langle \mathbf{M}\cdot {\mathbf{r}}_{\text{RFC}}\rangle}$$

where **M** is a coordinate transformation matrix that converts coordinates from the rotation gantry system $xyz$ to the fixed room system $XYZ$

3 $$\mathbf{M}=\left(\begin{array}{ccc}\text{cos}\varphi & 0& \text{sin}\varphi \\ 0& 1& 0\\ -\text{sin}\varphi & 0& \text{cos}\varphi \end{array}\right)$$

and ${\mathbf{r}}_{\text{RFC}}=\left({x}_{\text{RFC}},{y}_{\text{RFC}}\right)$ represents the positions of the RFC [18, 21] relative to the collimator axis ${\mathbf{C}}_{0}$. The $\langle \; \rangle$ in Eq. (2) denotes averaging over all possible gantry angles $\varphi$. The RFC, ${\mathbf{r}}_{\text{RFC}}$, can be split up into two components: a mechanical field centre (MFC) relative to the collimator axis ${\mathbf{r}}_{\text{MFC}}=\left({x}_{\text{MFC}},{y}_{\text{MFC}}\right)$ and an FS contribution, $\mathbf{d}$. By describing the RFC in the $xyz$-coordinate system with the origin at the centre of the line element defined by the collimator axis intersection point described previously in this section, i.e., at the mechanical isocentre, one can deduce from geometric considerations

4 $${\mathbf{r}}_{\text{RFC}}=\mathbf{d}+\frac{{{\ell}}_{\text{I}}}{{{\ell}}_{\text{BLD}}}\left({\mathbf{r}}_{\text{MFC}}-\mathbf{d}\right)$$

with ${\mathbf{r}}_{\text{MFC}}$ being defined at the midpoint of the BLD located at the distance ${{\ell}}_{\text{BLD}}$ from the FS. The quantity ${{\ell}}_{\text{I}}={{\ell}}_{\text{I}}\left(\varphi \right)$ is gantry angle dependent, while ${{\ell}}_{\text{BLD}}$ is considered a constant. Both ${\mathbf{r}}_{\text{MFC}}$, and the FSP $\mathbf{d}$**,** contribute to the gap between the radiation and mechanical isocentre, Eqs. (2)–(4). The RFC, ${\mathbf{r}}_{\text{RFC}}$, at the gantry angle $\varphi$ can be determined by averaging the position of the field centre for mutual opposing collimator angles, $\theta$, equally distributed over 360º. Similarly, ${\mathbf{r}}_{\text{MFC}}$, is understood as the average position of the geometric centre of a rectangular field relative to the collimator axis, ${\mathbf{C}}_{0}$, found as an average over the same collimator angles, $\theta$, as for ${\mathbf{r}}_{\text{RFC}}$. Furthermore, $\mathbf{d}$ is measured using the rigid phantom as described in this work, enabling the determination of ${\mathbf{r}}_{\text{MFC}}$, using Eq. (4). The mechanical field centre, ${\mathbf{r}}_{\text{MFC}}$, is an effect arising from the mechanical sag of the BLDs. If the sag of the BLD ${\mathbf{r}}_{\text{MFC}}\cong 0$ mm, the RFC deviation from the central axis is determined by **d** alone. By comparing Eqs. (1) and (4) with ${\mathbf{r}}_{\text{MFC}}=0$, it is found that ${\mathbf{r}}_{\text{RFC}}={\mathbf{C}}_{\text{BLD}}-{\mathbf{C}}_{0}$.

Assuming a phenomenological approach, both the FSP, $\mathbf{d}$**,** and the mechanical field centre, ${\mathbf{r}}_{\text{MFC}}$, can be modelled as periodic and sinusoidal functions over 360° of the form.

5 $$\mathbf{d}=\left({a}_{x}+{b}_{x}\text{sin}\varphi +{c}_{x}\text{cos}\varphi , {a}_{y}+{b}_{y}\text{sin}\varphi +{c}_{y}\text{cos}\varphi \right)$$

and

6 $${\mathbf{r}}_{\text{MFC}}=\left({s}_{x}\text{sin}\varphi ,{s}_{y}\text{cos}\varphi \right)$$

where ${a}_{x}, {b}_{x}, {c}_{x}, {a}_{y}, {b}_{y}, {c}_{y}$ are constants describing the FSP and ${s}_{x},{s}_{y}$ are constants describing the mechanical sag of the BLDs. The BLD sag, Eq. (6), assumes cardinal collimator angles angle $\theta$. For collimator angle 0° or 180°, ${s}_{x}$ represents the sag of the MLC and ${s}_{y}$ is the sag of jaws due to gantry arm bending, while for collimator angles $-90$° (270°) and 90°, it is the opposite. By inserting Eqs. (5)-(6) in Eqs. (2) and (4) and average over 360°, assuming ${{\ell}}_{\text{I}}$ being a constant, yields

7 $$\Delta \mathbf{I}=\frac{1}{2}\left(1-\frac{{{\ell}}_{\text{I}}}{{{\ell}}_{\text{BLD}}}\right)\left({c}_{x},2{a}_{y},-{b}_{x}\right)-\frac{1}{2}\frac{{{\ell}}_{\text{I}}}{{{\ell}}_{\text{BLD}}}\left(\text{0,0},{s}_{x}\right)$$

The quantity $\Delta \mathbf{I}$, as defined in Eq. (7), represents the shift between isocentre defined for the linac model in the TPS and the physical linac. The shift was found to be independent of ${s}_{y}$,${a}_{x},$ ${b}_{y}$, and ${c}_{y}$. The ideal situation for the isocentre position would occur when $\left|\Delta \mathbf{I}\right|=0$.

### EPID image acquisition

Different parameters are configured to control the EPID image acquisition in the iViewGT™ software’s sri.ini initialisation file [31]. A threshold parameter, PortBeamOnThreshold (default 300), is set to prevent imaging during beam ramp-up, and a PostBeamOff parameter determines the frames read out after beam off (default 3) [32, 33]. In this study, we followed Elekta’s recommendation and used the default values for all frame acquisitions. The final image for each beam segment was obtained by averaging the entire set of frames.

The number of usable frames ${N}_{f,U}$ acquired at $U$ monitor units (MU) with a pulse repetition frequency (PRF) of ${\nu }_{p}$ was observed to follow.

8 $${N}_{f,U}=\left(\frac{{\alpha }_{E}}{{\nu }_{p}}\right)U$$

where ${\alpha }_{E}$ is an energy and panel-specific parameter that characterises the frames generated per second and per MU. Equation (8) arises from the time-based nature of the EPID image acquisition process, where the number of usable frames, ${N}_{f,U}$, is directly proportional to the ‘‘beam on’’ time $\Delta t$ of the MV. Since $\Delta t$ is proportional to $U/{\upsilon }_{p}$ the relationship between ${N}_{f,U}$ and ∆t explains Eq. (8). The assumed total number of frames,${N}_{f}$, forming an image is provided as

9 $${N}_{f}={N}_{f,U}+{N}_{f,0}$$

with ${N}_{f,0}$ representing the number of PostBeamOff frames. At low values of $U$, the counts of usable frames could be as low as one (1). Hence, it is assumed that iViewGT only displays the usable frame number during beam on, i.e., ${N}_{f,U}$, and the three frames acquired after beam off, i.e., ${N}_{f,0}$, are not counted as usable frames, Eq. (9).

## Results

### EPID acquisition with AL and AP panels

The value of ${\alpha }_{E}$ was estimated by counting the number of usable frames listed on the iViewGT monitor during the acquisition of 1000 MU at a nominal dose rate. The corresponding results are presented in Table 3. Remarkably, the ${\alpha }_{E}$ parameter was found to be independent of the field size, dose rate and presence of material that attenuates the beam.

**Table 3:** The number of frames per MU and PRF, α_E_, was measured for different energies and EPID panels. The data in the table were generated later than most FSP measurements. In addition, linac B and D were out of clinical service at the time of measurements

Linac	Panel type	6 MV α_E_ (MU s)^−1^	18 MV α_E_ (MU s)^−1^	6 MV FFF α_E_ (MU s)^−1^
A	AL	141	47	–
C	AP	133	45	50
E	AL	149	41	–
F	AL	119	51	–
G	AP	132	58	47
H	AP	139	52	50

It is worth noting that the time of acquisition for one frame (${T}_{f}$) is 320 ms (AL panel) and 128 ms (AP panel) [31]. However, despite the different acquisition times, the values in Table 3 indicate an apparent equality in the acquisition times for AL and AP panels. This suggests that the handling of frames is dissimilar for the two panel models. Specifically, AP panels average each set of three frames into one frame [31], effectively making the image acquisitions for both panel types nearly equal. The main distinction is that AP panels allow for higher dose rates compared to AL panels, thereby enabling EPID images at the 6 MV FFF nominal dose rate.

For nominal dose rates with a PostBeamOff value of 3, it can be estimated from Table 3 and Eqs. (8)-(9) that a 10 MU beam will acquire approximately 6–7 frames, 5–6 frames and 4 frames for 6 MV, 18 MV and 6 MV FFF beams, respectively. The variation in the number of frames acquired is influenced by the beam on time $\Delta t$. The time $\Delta t$ to deliver a beam of $U$ monitor units depends in particular on the energy and also the dose rate performance of the linac. This explains the discrepancies in the value of in Table 3.

### Focal spot position data based on the rigid ball-bearing phantom

The data presented in this study consist of 147 series of images acquired on eight linacs, resulting in a total of 24,843 images (147 × 169). Among these, 106 series were acquired with the AL panel, and 41 series were acquired with the AP panel. All eight linacs were in clinical service during the measurement period, which was three years and four months.

The acquired data consist of 135 series with a CCW rotation and 12 series with a CW rotation. Additionally, there were 45 series with 6 MV, 43 series with 18 MV and 59 series with 6 MV FFF. For all eight linacs, at least three CCW series were taken. The FSP in the *x* and *y* coordinate system was calculated and plotted. Out of the 147 series, 129 were acquired with 10 MU, 9 series with 3 MU, and another 9 with 50 MU per image. The PRF was set to the nominal default value (${\nu }_{p}$= 400 Hz for 6 MV and 6 MV FFF) and (${\nu }_{p}$= 200 Hz for 18 MV). However, the 6 MV FFF data consists of 34 series acquired at 100 Hz and 25 series at 400 Hz.

**Fig. 2:**
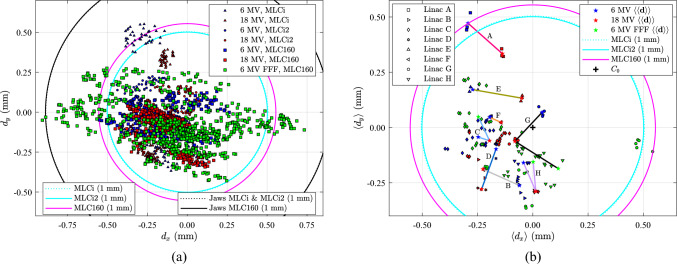
Plots representing all data of FSP. The circles represent the FSP resulting in a 1 mm CAP offset relative to the collimator axis measured by different BLDs. (**a)** FSP for each gantry angle and all linacs, $\mathbf{d}$. The number of points 13 × 143 = 1859. (**b**) The data points represent the average position of all gantry angles for each series, energy and for all linacs (A to H). The mean of $\langle \mathbf{d}\rangle$ across each linac energy, $\langle \langle \mathbf{d}\rangle \rangle$, is marked with a star

An overview of the FSP measurements of all the linacs is displayed in Fig. 2. The FS distance, $|\mathbf{d}|$, from the collimator axis resulting in a 1 mm central axis deviation from the collimator axis at isocentre distance defines a circle. In the figure, the circles representing each of the BLDs (MLCi, MLCi2, MLC160 and jaws) are drawn. The radius of the circle is obtained from Eq. (1) by inserting $\left|{\mathbf{C}}_{\text{BLD}}-{\mathbf{C}}_{0}\right|=1$ mm, ${{\ell}}_{\text{I}}$ = 100 cm, and the values of ${{\ell}}_{\text{BLD}}$ defined as the distance from the FS to the midpoint of the BLD. The midpoint of the BLDs of the different radiation heads is shown in Table 2 as well as the geometrical data behind the midpoint estimation.

In Fig. 2a, data points of all linacs are shown for all gantry angles and energies. Some data points lie outside the limit of 1 mm, especially 6 MV FFF data in the *x*-direction. In Fig. 2b, the data points $\langle \mathbf{d}\rangle =\left(\langle {d}_{x}\rangle ,\langle {d}_{y}\rangle \right)$ represent the FSP averaged over 360° degree gantry rotation for each energy and linac. The mean of $\langle \mathbf{d}\rangle$, i.e., $\langle \langle \mathbf{d}\rangle \rangle$, is shown in Fig. 2b for each linac with a star symbol indicating energy at the endpoint of line elements connecting the energies of the linac. Most variation is seen for the 6 MV FFF beams. Linac A (MLCi) 6 MV was found to be outside the 1 mm range. Some measurements for linac G (MLC160) with 6 MV FFF were close to the 1 mm limit.

The linacs A, B, D, E and F were investigated over a short time period (a few days). The other linacs (C, G, and H) were investigated over a more extended period of time, especially for 6 MV FFF (over 2.7 years). Long-time effects (over 3.5 years) on 6 MV and 18 MV were only considered for linac C. A more extensive spread is seen for linac C (6 MV and 18 MV) compared to the other linacs, which is an indication of the time drift of linac C, Fig. 2b. Furthermore, the more extensive spread of 6 MV FFF (linac C, G, and H), Fig. 2b, also indicates drift over time.

**Fig. 3:**
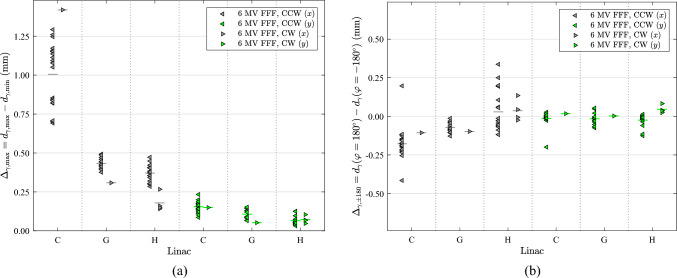
Plots of maximum and start/stop ranges of 6 MV FFF beam data for linacs C, G, and H are shown as scatter plots for CCW and CW gantry rotations. The vertical lines indicate the mean values of each series. The $\gamma$ parameter is either $x$ or . (**a**) Maximum range ${\Delta }_{\gamma ,\text{max}}$. (**b**) Start/stop range ${\Delta }_{\gamma ,\pm 180}$

**Fig. 4:**
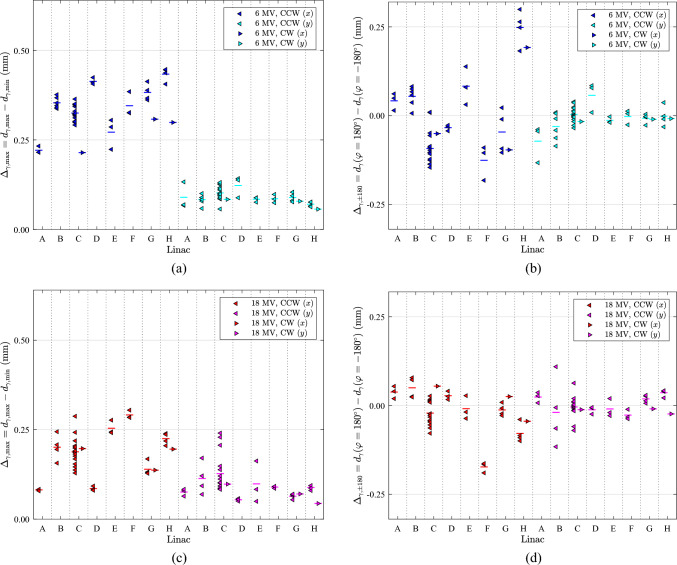
Plot of maximum and start/stop ranges of 6 MV and 18 MV beam data for linacs A-H are shown as scatter plots for CCW and CW gantry rotations with $\gamma$ being either $x$ or $y$. The vertical lines indicate the mean values of each series. (**a)** Maximum range ${\Delta }_{\gamma ,\text{max}}$ of 6 MV. (**b)** Start/Stop range ${\Delta }_{\gamma ,\pm 180}$ of 6 MV. (**c)** Maximum range for 18 MV. (**d**) Start/stop range ${\Delta }_{\gamma ,\pm 180}$ for 18 MV

The stability of the FSP is characterised by two parameters as the value of the largest FSP difference over a 360° gantry rotation (${\Delta }_{x,\text{max}},{\Delta }_{y,\text{max}}$), and the difference between gantry angles –180° and 180° (${\Delta }_{x,\pm 180},{\Delta }_{y,\pm 180}$) calculated in both the *x*- and *y*-direction. Low spread in the data indicates superior stability, while values close to zero signify optimal performance. The FSP stability for different energies is shown in Fig. 3 for 6 MV FFF and in Fig. 4 for 6 MV and 18 MV. The data is presented in scatter plot forms for CCW and CW rotations.

The ${\Delta }_{x,\text{max}}$ value appears to be largest for linac C, 6 MV FFF in Fig. 3a, while linacs G and H are at a lower and equal level. ${\Delta }_{y,\text{max}}$ shows a similar but less pronounced pattern in Fig. 3a. The ${\Delta }_{x,\pm 180}$ values in Fig. 3b have a more extensive spread for linac C and H than for G. For ${\Delta }_{y,\pm 180}$ the linacs are alike. The mean values of ${\Delta }_{x,\pm 180}$ deviate more than ${\Delta }_{y,\pm 180}$ from zero.

In Fig. 4a, c, the ${\Delta }_{x,\text{max}}$ values are approximately 0.25 mm larger for 6 MV than for 18 MV. The ${\Delta }_{y,\text{max}}$ are equal with a tendency of a larger spread of the 18 MV data. In Fig. 4b, d a larger spread in the ${\Delta }_{x,\pm 180}$ data are seen for 6 MV than 18 MV. The mean of linac H for 6 MV and linac F for 18 MV deviates most from the other linacs, as seen in Fig. 4b, d.

The linac C, G and H data for 6 MV FFF and linac C for 6 MV and 18 MV were acquired over three years and four months. This might explain the larger spread in the data of these linacs and energies. Both ${\Delta }_{y,\text{max}}$ and ${\Delta }_{y,\pm 180}$ seem more stable with less variation than the corresponding *x*-component. The 18 MV beam has the best performance, which might be due to the more significant sensitivity in the beam steering for collimator off-axis beams caused by the strong beam filtration of 18 MV. For all energies, it seems that ${\Delta }_{y,\text{max}}<{\Delta }_{x,\text{max}}$ and $\left|{\Delta }_{y,\pm 180}\right|<\left|{\Delta }_{x,\pm 180}\right|$ are fulfilled in most cases. Changes in linac performance over time due to temperature, temperature gradients, use, and attrition of core components involved in beam generation might impact the stability of the FSP.

Using the simplified model, Eq. (5), one achieves ${\Delta }_{\gamma ,\text{max}}=2\sqrt{{b}_{\gamma }^{2}+{c}_{\gamma }^{2}}$ and ($\gamma =x \text{or} y$). To minimise the range ${\Delta }_{\gamma ,\text{max}}$ requires small values of the gantry angle-dependent amplitudes ${b}_{\gamma }$ and ${c}_{\gamma }$. Beam optimisation is a challenging duty involving compromises accepting ${\Delta }_{\gamma ,\text{max}}\ne 0$ mm.

**Fig. 5:**
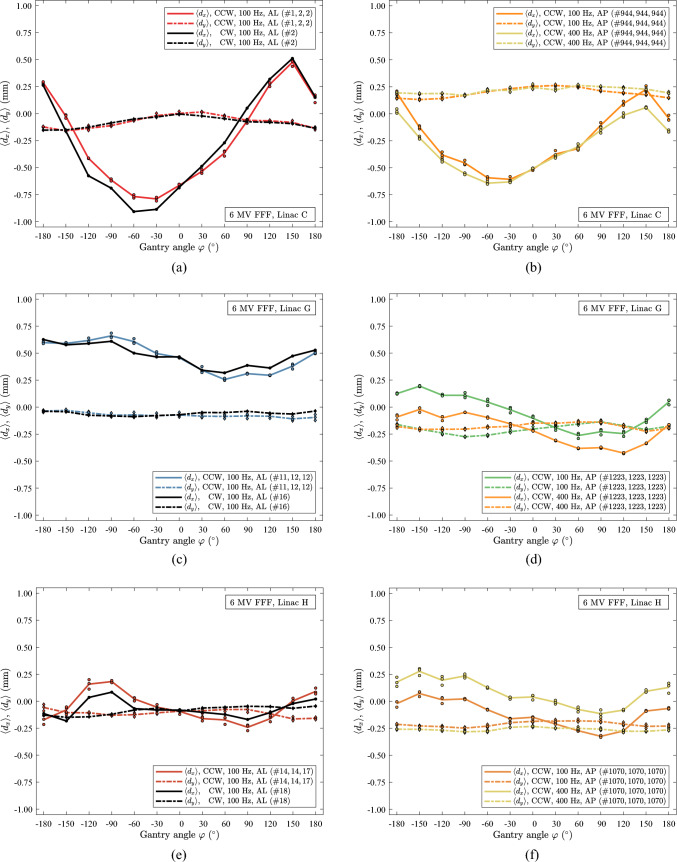

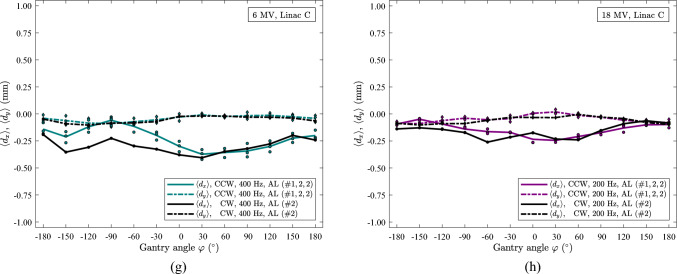
Beam data for linac C, G and H, 6 MV FFF, 10 MU for each segment. (**a)** linac C, AL panel, CW and CCW data. (**b)** linac C, AP panel, 100 and 400^†^ Hz data. (**c)** linac G, AL panel, CW and CCW data. (**d)** linac G, AP panel, 100 and 400^†^ Hz data. (**e)** linac H, AL panel, CW and CCW data. (**f)** linac H, AP panel, 100 and 400 Hz data. †: reproduction [16], AP panel data acquired at 400 Hz in CCW rotation on linac C (#944, 944, 944) and G (#1223, 1223, 1223). (**g)** linac C, AL panel, 400 Hz, CW and CCW. (**h)** linac C, AL panel, 200 Hz, CW and CCW. In the figures, the $x$-axis orientation has been shifted to be consistent with the $x$-axis orientation in previous work [16]

In Fig. 5 both scans with CW and CCW rotations, as well as dose rate dependency of 6 MV FFF for three different linacs are presented as a function of the gantry angle $\varphi$. In Fig. 5a, c, e, the CW (one scan) and CCW (three scans) data coincide and intersect at gantry 0°. For negative gantry angles, the CW scan tends to be above the CCW scan, while for positive gantry angles, it tends to be below. The curves exhibit similar shapes for both CW and CCW rotation. The data shown in Fig. 5a, c, e represent scans conducted within three days for each linac and energy, collected and plotted accordingly. A dose rate dependency was observed, as different scan results were obtained for a PRF of 100 and 400 Hz of 6 MV FFF beams delivered on the same day, as shown in Fig. 5b, d, f. The dependency is most pronounced for the $x$-values, particularly for linac H, and to a lesser extent for linac C. Linac G and H, show a constant shift in the $x$-values, which is not the case for linac C. Additionally, minor changes occur in the $y$-values for the three linacs.

In Fig. 5, scans with CW (one scan) and CCW (three scans) gantry rotation of 6 MV, Fig. 5g and 18 MV, Fig. 5h are shown as a function of gantry angle $\varphi$. The dependence on angle is small. A small shift is seen in the $\langle {d}_{x}\rangle$ from zero while $\langle {d}_{y}\rangle \cong 0.00$ mm. A minor CW and CCW dependency is seen in $\langle {d}_{x}\rangle$, Fig. 5g.

**Fig. 6:**
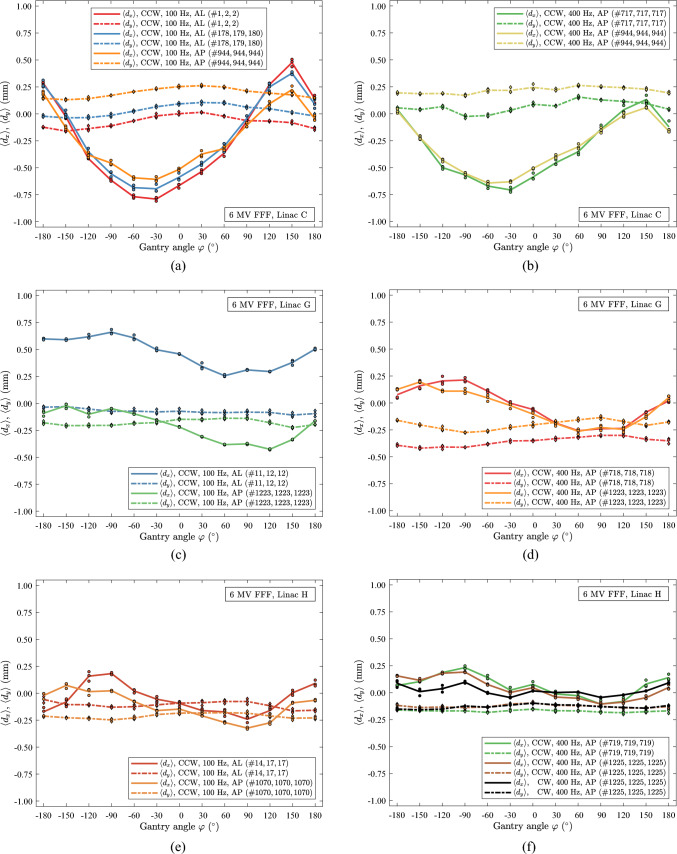
Data of 6 MV FFF for linac C, G and H. All data are with 10 MU for each segment, CCW rotation. (**a)** Linac C, 100 Hz. (**b)** Linac C, 400^†^ Hz. (**c)** Linac G, 100 Hz. (**d)** Linac G, 400^†^ Hz. (**e)** Linac H, 100 Hz. (**f)** Linac H, 400^†^ Hz. †: reproduction [16], AP panel data acquired at 400 Hz in CCW rotation on linac C (#944, 944, 944), G (#1223, 1223, 1223) and linac H (#1225, 1225, 1225). In the figures, the $x$-axis orientation has been shifted to be consistent with the $x$-axis orientation in previous work 16

Figure 6 displays FSP scans of 6 MV FFF as a function of gantry angle, $\varphi$, collected over an extended period and grouped into 100 Hz (AL and AP panels) and 400 Hz (only AP panel) for linac C, G and H. A substantial shift in the $x$-component for 100 Hz scans of linac G is found, Fig. 6c, while a change in the curve shape is more pronounced for linacs C and H, Fig. 6a, e. The $x$ data acquired at 400 Hz seem to be only slightly affected over time for the three linacs, Fig. 6b, d, e. The $y$-components tend to undergo a shift over time, Fig. 6a, c, e. At 400 Hz, *y* shows a shift for linac C and G, Fig. 6b, d; while a shift in linac H is not obvious, Fig. 6f.

In Fig. 6f, CW and CCW data acquired three times on the same day are shown. Moreover, the data in Fig. 6f follow the same feature discussed for the data shown in Fig. 5e. A slight shift in the $x$ data is seen over time, Figs. 5e, 6f.

**Fig. 7:**
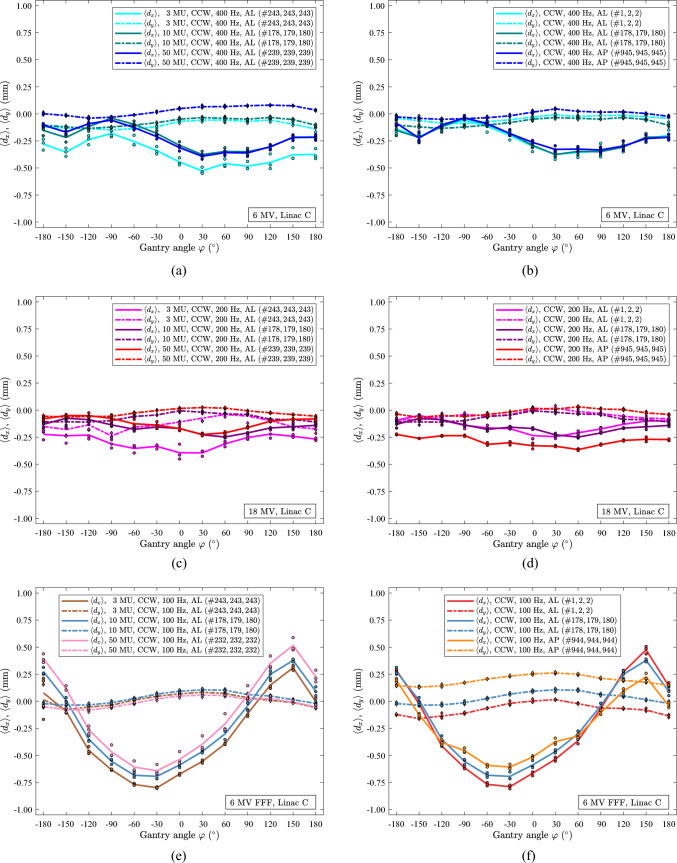
Data of linac C for 6 MV, 18 MV and 6 MV FFF. All scans were carried out in CCW rotation. (**a)** scans of 6 MV beams at 400 Hz with 3 MU, 10 MU and 50 MU. (**b)** scans with 6 MV beams at 400 Hz with 10 MU before and after the scans in (**a**). (**c)** scans of 18 MV beams at 200 Hz with 3 MU, 10 MU and 50 MU. (**d)** scans with 18 MV beams at 200 Hz with 10 MU before and after the scans in (**c**). (**e)** scans of 6 MV FFF beams at 100 Hz with 3 MU, 10 MU and 50 MU. (**f)** scans with 6 MV FFF beams at 100 Hz with 10 MU before and after the scans in (**e**)

In Fig. 7, the performance of linac C for 6 MV, 18 MV and 6 MV FFF as a function of gantry angle, $\varphi$, is shown. Fig. 7a, c, e exhibit scans with a beam exposure of 3, 10, and 50 MUs at each gantry angle. Notably, these scans were conducted on different days. To assess long-term beam stability, a selected 10 MU data set for each energy is used as a reference, see Fig. 7a,b, 7c,d and 7e,f. The shape of the curves, $\langle {d}_{x}\rangle$ and $\langle {d}_{y}\rangle$, in Fig. 7a, c, e appear mostly independent of the number of MUs used. The shifts of the curves are less than 0.25 mm. The translations of $\langle {d}_{x}\rangle$ and $\langle {d}_{y}\rangle$ are in the positive direction for an increase from 3 to 50 MU for 6 MV and 18 MV. For 6 MV FFF, the translation in $\langle {d}_{x}\rangle$ is in the positive direction, while the shift in $\langle {d}_{y}\rangle$ is minor and in the negative direction. Over a period of 945 days, Fig. 7b, d, f, significant drift is observed in the *y* data for 6 MV, Fig. 7b, *x* for 18 MV, Fig. 7d and *x* and *y* data for 6 MV FFF, Fig. 7f. No significant difference is observed between the data acquired with the two panels (AL and AP) because the data spread is similar, and the curve shapes are comparable for both panels. However, a noticeable shift in the curves is observed, likely due to the scans being acquired on different days.

### PTW STARCHECK phantom

**Fig. 8:**
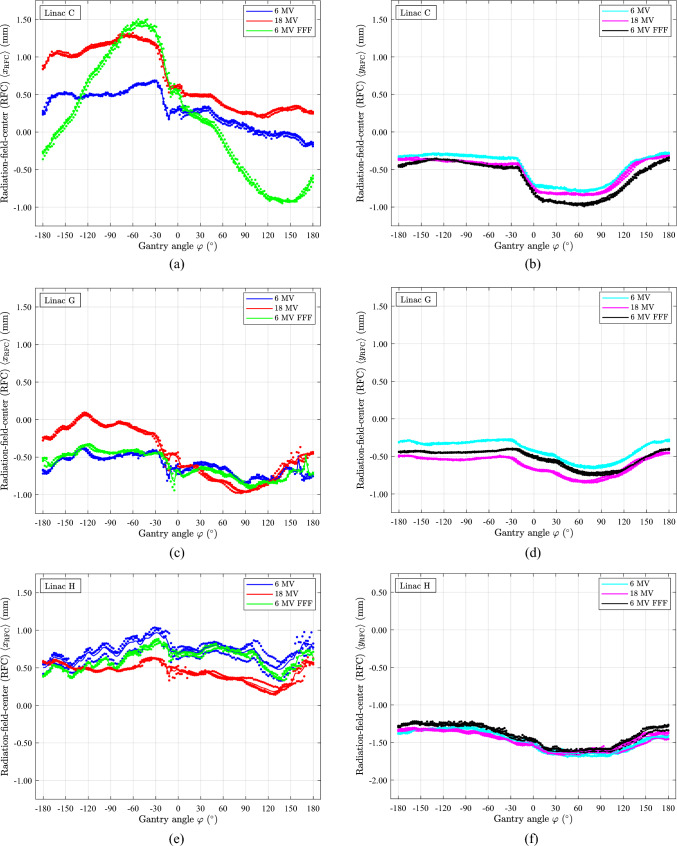
The figure displays the mean radiation-field-centre (RFC) as the results of STARCHECK measurements during CCW gantry rotation at nominal dose rates. The sensitive detectors were at SDD = 100 cm and had a build-up of 1.85 cm. The mean RFCs $\langle {x}_{\text{RFC}}\rangle$ and $\langle {y}_{\text{RFC}}\rangle$ are shown for three different linacs all with the energies 6 MV, 18 MV, and 6 MV FFF. The data points represent three series of measurements, while the fully drawn line shows the mean value of three measurements. Linac C (Day #1840), linac G and H (Day #1832). (**a)** $\langle {x}_{\text{RFC}}\rangle$, linac C. (**b)** $\langle {y}_{\text{RFC}}\rangle$, linac C. (**c)** $\langle {x}_{\text{RFC}}\rangle$, linac G. (**d)** $\langle {y}_{\text{RFC}}\rangle$, linac G. (**e)** $\langle {x}_{\text{RFC}}\rangle$, linac H. (**f)** $\langle {y}_{\text{RFC}}\rangle$, linac H

Figure 8 presents PTW STARCHECK measurements on linac C, G, and H, showcasing the RFC location in the CR ($x$) and IN ($y$) plane directions under 360° gantry rotation of three CCW gantry rotations for each energy. It is worth noting that the BLDs and the rack holding the STARCHECK phantom both exhibit sag during gantry rotation [7]. Despite appearing rigid and being mounted to the radiation head of the linac without geometric hysteresis, the STARCHECK phantom rack still shows sag.

The mean RFC, $\langle {x}_{\text{RFC}}\rangle$, in Fig. 8a for 6 MV FFF and the $\langle {d}_{x}\rangle$ determined using the rigid ball-bearing phantom, Fig. 8b, exhibit similarities but with opposite signs. This observation aligns with Eq. (1), where it is evident that the FSP **d** and the RFC ${\mathbf{r}}_{\text{RFC}}$ have different signs and are scaled differently from one another. A comparable effect is observed for $\langle {y}_{\text{RFC}}\rangle$ in Fig. 8d compared to Fig. 8d for the 6 MV FFF beam. As for Fig. 8e, it is unclear whether 6 MV FFF shares the same feature as seen in Fig. 8f. However, for the other energies and directions, Fig. 8b, d, f, the relation to the ball-bearing phantom data is less certain. This uncertainty may arise from the larger sag effects from the STARCHECK holding rack and BLDs, which could outweigh the FSP offset’s impact and consequently diminish the effect.

The spread of the ${x}_{\text{RFC}}$ and ${y}_{\text{RFC}}$ data in each series appears relatively small, with the smallest spread observed in the $y$ direction, likely due to fewer effects from gravity. However, larger spreads in ${x}_{\text{RFC}}$ are evident in Fig. 8e, which may be related to the geometric hysteresis of the attached rack. Interestingly, the hysteresis effects seem different for the three energies, indicating unstable beam steering, primarily for 6 MV, as a more likely cause. Additionally, there is a distinct shift relative to the origin, likely associated with the alignment of the phantom to the linac cross-wire at gantry 0° for each linac setup. The setup was deemed acceptable according to the cross-wire, and the same phantom position relative to the rack was maintained for the three setups on the three linacs.

The mean RFC $\langle {x}_{\text{RFC}}\rangle$ of 6 MV FFF, linac C, Fig. 8a, deviates from linacs G and H, Fig. 8c, e. The mean RFC $\langle {x}_{\text{RFC}}\rangle$ appears similar for 6 MV and 6 MV FFF for linacs G and H, Fig. 8c, e. The mean RFC $\langle {x}_{\text{RFC}}\rangle$ of 18 MV, Fig. 8a, c, e, demonstrates similarities in the gantry angle dependency as seen for 6 MV measured at the same linac. The mean RFC, $\langle {y}_{\text{RFC}}\rangle$, Fig. 8b, d, f, exhibits minor variations with gantry angle, and the general structure of the gantry dependency remains similar for all energies and linacs.

**Fig. 9:**
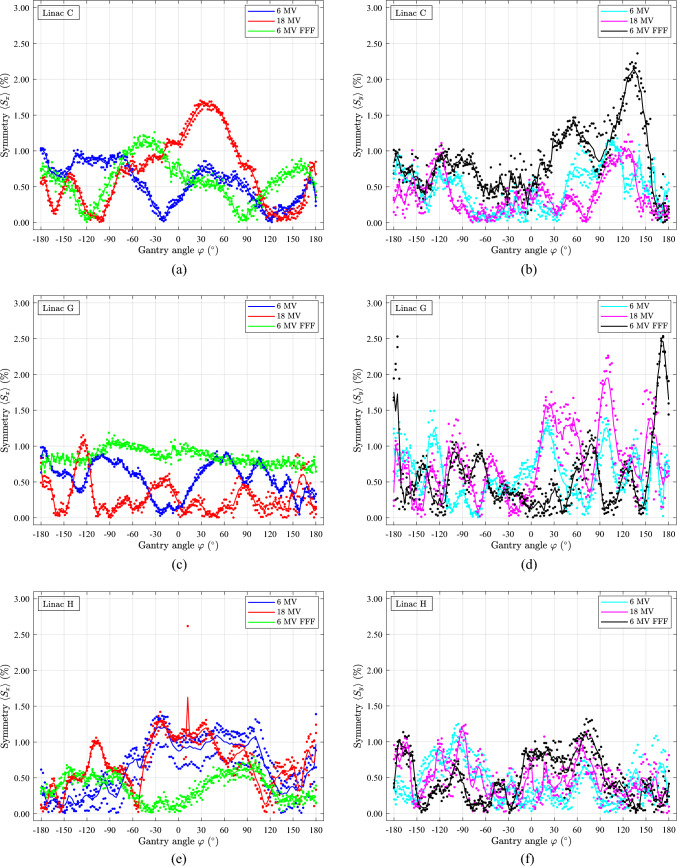
Symmetry calculations of the beams shown in Fig. 9. All gantry rotations are in the CCW direction. Each data point displays one measurement, while the fully drawn curve represents the average of three measurements. (**a)** $\langle {S}_{x}\rangle$, linac C. (**b)** $\langle {S}_{y}\rangle$, linac C. (**c**) $\langle {S}_{x}\rangle$, linac G. (**d)** $\langle {S}_{y}\rangle$, linac G. (**e)** $\langle {S}_{x}\rangle$, linac H. (**f)** $\langle {S}_{y}\rangle$, linac H

The mean symmetry data $\langle {S}_{x}\rangle$ and $\langle {S}_{y}\rangle$ in Fig. 9 are based on the same data as the RFC data shown in Fig. 8. From Fig. 9a, c, e it is observed that $\langle {S}_{x}\rangle <1.8\%$, while from Fig. 9b, d, f one has $\langle {S}_{y}\rangle <2.6\%$. Peaks and variations are noticeable in the $\langle {S}_{x}\rangle$ and $\langle {S}_{y}\rangle$ data as a function of gantry angle, but for most of a gantry 360° rotation, the symmetries $\langle {S}_{x}\rangle$ and $\langle {S}_{y}\rangle$ are within < 1%. There appears to be no strong correlation between RFC and symmetry when comparing data from Fig. 8 with Fig. 9. However, the spread in ${x}_{\text{RFC}}$, Fig. 8e, of linac H also results in a spread of ${S}_{x}$, Fig. 9e. The spread in RFC is transferred to the calculations of symmetry since the symmetry calculations use the RFC at (${x}_{\text{RFC}},{y}_{\text{RFC}})$ as a reference position for symmetry calculations.

The tolerance of symmetry for X-rays is $\pm 1$% from baseline [1]. During commissioning, the symmetry data were measured in a water tank as a profile scan with a single detector at gantry 0° in the depths 5, 10 and 20 cm with a source-surface distance (SSD) of 90 cm. The commissioned beams’ symmetry baseline was $\approx 0$% based on the previously described symmetry definition measured at a depth of 10 cm and SSD = 90 cm. If the baseline was set using the STARCHECK phantom at gantry 0° mounted to the radiation head, a baseline of $\approx 0$% at a PMMA depth of 1.85 cm with an SSD of 98.85 cm would have been achieved at the time of commissioning. The symmetries measured using STARCHECK at gantry 0° are almost within the tolerance of $\pm 1$%, Fig. 9. However, it would be possible to adjust this value of symmetry at gantry 0° to a lower value using the STARCHECK phantom to meet the tolerance in Fig. 9. Such an adjustment might impact the symmetry measured at water depth 10 cm and SSD 90 cm. However, whether a water tank measurement carried out on the same day as the STARCHECK measurements would exhibit the same feature on symmetry is unknown.

Notably, the data’s spread from three successive measurements is small in both Figs. 8, 9, confirming the reliability of the measurement method using the STARCHECK phantom.

### SNC ArcCHECK phantom

**Fig. 10:**
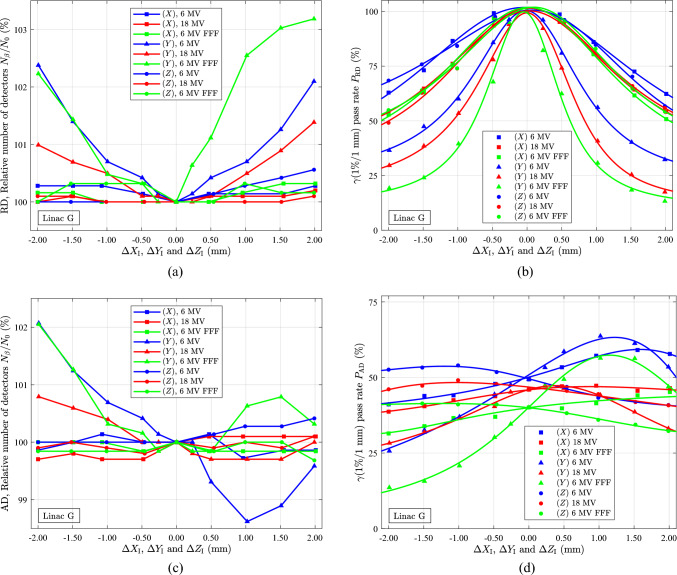
The relative number of detectors in the analyses ${N}_{\beta }/{N}_{0}$ and the RD and AD gamma ($\gamma$) pass rates using the criteria ($\Delta {d}_{\text{M}}$, $\Delta {D}_{\text{M}}$) = (1%, 1 mm). The pass rates are shown for three dose plan deliveries at 6 MV, 18 MV, and 6 MV FFF measured using the ArcCHECK phantom on linac G (Day #1709,1710). Each data point represents one measurement. The smooth curves are based on the fitting function, Eq. (10). (**a)** The relative number of detectors involved in the pass rate analysis ${N}_{\beta }/{N}_{0}$ with ${N}_{0}$ at (0,0,0) being 715, 1011, and 628 for 6 MV, 18 MV, and 6 MV FFF, respectively. (**b)** Pass rates ${P}_{\text{RD}}$ for three orthogonal shifts in the $X,Y,Z$-directions as $(\Delta {X}_{\text{I}},\text{0,0}), (0,\Delta {Y}_{\text{I}},0),$ and $(\text{0,0},\Delta {Z}_{\text{I}}).$ (**c)** The relative number of detectors involved in the pass rate analysis ${N}_{\beta }/{N}_{0}$ with ${N}_{0}$ at (0,0,0) being 724, 1011, 634 for 6 MV, 18 MV and 6 MV FFF, respectively. (**d)** Pass rates ${P}_{\text{AD}}$ for three orthogonal shifts in the $X,Y,Z$-directions as $(\Delta {X}_{\text{I}},\text{0,0}), (0,\Delta {Y}_{\text{I}},0),$ and $(\text{0,0},\Delta {Z}_{\text{I}})$

In Fig. 10 and 11, deviations in the number of detectors and pass rates based on gamma ($\gamma$) analyses [26, 34] are shown as a function of position shifts ($XYZ$) relative to the reference position of the ArcCHECK phantom. The reference position refers to the room laser match to the phantom’s isocentre (I) markings. All measurements were conducted on linac G.

Pass rates were calculated based on the number of detectors with the relative dose (RD) differences between the TPS calculated dose delivery and the measured dose delivery to the phantom. The normalisation was relative to the maximum measured and maximum calculated doses in the TPS plan, respectively. The $\gamma$ RD pass is based on a user-defined percentage maximum dose error allowed ($\Delta {D}_{\text{M}}$), $\Delta {D}_{\text{M}}$ = 1%, and a user defined distance criterion limit ($\Delta {d}_{\text{M}}$), $\Delta {d}_{\text{M}}$ = 1 mm meeting the lower-dose threshold (LDT) criteria relative to all detectors.

In Fig. 10a, the number of detectors measuring relative doses above the threshold increases for shifts along the $\Delta {Y}_{\text{I}}$ direction, while showing almost no change in the $\Delta {X}_{\text{I}}$ and $\Delta {Z}_{\text{I}}$ directions. This effect is expected to have a negligible impact on the pass rate ${P}_{\text{RD}}$, as shown in Fig. 10b. However, the pass rate ${P}_{\text{RD}}$ is sensitive to small shifts relative to the reference position, particularly in the $\Delta {Y}_{\text{I}}$ direction. The sensitivity was also found to be energy-dependent, mainly in the $\Delta {Y}_{\text{I}}$ direction. Shifts in $\Delta {Y}_{\text{I}}$ < 0.25 mm can be detected for all energies, whereas shifts in $\Delta {X}_{\text{I}}$ and $\Delta {Z}_{\text{I}}$ > 0.5 mm are detectable.

In Fig. 10, the dose calculated in the TPS system is compared with the measured dose delivery. The relative number of detectors above the threshold, as depicted in Fig. 10a, seems similar for negative values of $\Delta {X}_{\text{I}}$, $\Delta {Y}_{\text{I}}$ and $\Delta {Z}_{\text{I}}$, as shown in Fig. 10c, but differs, especially for positive $\Delta {Y}_{\text{I}}$ values. Fig. 10b, d displays the relative and absolute dose pass rates obeying the criteria ($\Delta {D}_{\text{M}}$, $\Delta {d}_{\text{M}}$) = (1%/1 mm). The measured dose and the TPS calculated dose are normalised to the maximum value in the TPS plan, i.e., global normalisation [35, 36]. The shifts in the $\Delta {Y}_{\text{I}}$ axis have the most significant effect on the pass rate, with a maximum of around $\Delta {Y}_{\text{I}}$=1.22 mm for 6 MV, $\Delta {Y}_{\text{I}}$=1.15 mm for 6 MV FFF, and $\Delta {Y}_{\text{I}}$=0.39 mm for 18 MV. The maximum pass rate ${P}_{\text{AD}}$ occurs for all energies around $-$ 1.0 mm for $\Delta {Z}_{\text{I}}$ and in the range 0.5–2.0 mm for $\Delta {X}_{\text{I}}$. The shift in $\Delta {Y}_{\text{I}}$ aligns with observations made over the years for many patient plan tests in our department using the ArcCHECK phantom. The shift was calculated in the SNC software and was frequently observed for 6 MV and 6 MV FFF plan energies and all Agility linacs. 

The pass rates ${P}_{\text{RD}}$ and ${P}_{\text{AD}}$ from the gamma analyses were found to be fitted by an exponential function of the form.

10 $${P}_{\alpha ,\beta }={C}_{\beta }\text{ exp}\left(\frac{-{\left({\Delta \beta }_{\text{I}}-{\mu }_{\beta }\right)}^{2}}{2{\sigma }_{\beta }^{2}\left[{A}_{\beta }{\left(\Delta {\beta }_{\text{I}}\right)}^{2}+{B}_{\beta }\Delta {\beta }_{\text{I}}+1\right]}\right)\alpha =\text{RD or AD}; \beta =X,Y,Z$$

where ${\mu }_{\beta }$, ${\sigma }_{\beta }$,${A}_{\beta }$,${B}_{\beta }$, and ${C}_{\beta }$ are fitting parameters. When,${A}_{\beta }={B}_{\beta }=0$, the fitting function simplifies to the Gaussian normal distribution with ${\mu }_{\beta }$ and ${\sigma }_{\beta }^{2}$ representing the mean and variance parameters, respectively. The parameters${A}_{\beta }$, and ${B}_{\beta }$ must satisfy the condition ${A}_{\beta }{(\Delta {\beta }_{\text{I}})}^{2}+{B}_{\beta }\Delta {\beta }_{\text{I}}+1>0,$ which holds if.

11 $${A}_{\beta }-{\left(\frac{{B}_{\beta }}{2}\right)}^{2}>0$$

The maximum pass rate occurs at.

12 $${\Delta \beta }_{\text{I}}={\mu }_{\beta }$$

and minimum at.

13 $${\Delta \beta }_{\text{I}}=-\frac{{B}_{\beta }{\mu }_{\beta }+2}{{B}_{\beta }+2{A}_{\beta }{\mu }_{\beta }}$$

A comparison between ${P}_{\text{RD}}$, Fig. 10b, and ${P}_{\text{AD}}$ Fig. 10d shows that the AD method is less sensitive to the phantom position than RD. The peaks of the AD data are broadened and located differently compared to the RD data, but in both cases, they are fitted well by the function, Eq. (10). Our analyses cannot clarify how the peak positions in the pass rates ${P}_{\text{RD}}$ the ${P}_{\text{AD}}$ can be interpreted, Fig. 10b, d . A movement of the phantom towards a higher pass rate indicates that the calculated TPS dose distribution better matches the measured dose distribution. However, it is essential to note that a movement towards a higher pass rate may not necessarily equate to a better match between the TPS plan isocentre and the linac isocentre. 

The SNC software has a build-in optimisation algorithm that calculates the $Y$ direction shift and phantom rotation along the phantom cylindrical axis to achieve the highest pass rates of ${P}_{\text{RD}}$ and ${P}_{\text{AD}}$. This functionality was not used in the present work. In clinical practice, the evaluation parameters of ${P}_{\text{AD}}$ was set to $(\Delta {D}_{\text{M}}, \Delta {d}_{\text{M}})=($3%/3 mm) and ${P}_{\text{AD}}\ge 95\%$. To have stronger sensitivity, the settings $(\Delta {D}_{\text{M}}, \Delta {d}_{\text{M}})=($1%, 1 mm) was used in this work.

## Discussion

### Rigid ball-bearing phantom

The most informative measure of the FSP relative to the collimator axis would be to detect the position for each linac pulse. However, the method presented in the study measures the FSP as an average position of multiple linac pulses, which has limitations in providing detailed information about each pulse. The assumed FSP stability at various collimator angles for a fixed gantry angle may not be entirely accurate, as a possible drift of the FSP for each linac pulse during acquisition could occur.

The determination of the FSP at one gantry angle was based on 13 EPID images acquired at 13 collimator angles, with the choice of 13 angles instead of three to ensure a robust method and avoid blurring of the ball-bearing appearance due to potential FSP drift during acquisition.

The FSP data as a function of the gantry angle showed some notable general features. Measurements repeated within a few days exhibited consistency with a small spread, while measurements repeated over larger time spans displayed more substantial differences. The observed slight displacement in ${d}_{x}$ or ${d}_{y}$, or both, between early and late measurements, is attributed to the wear of major linac components, such as the gun filament and the magnetron.

A dose rate effect for 6 MV FFF beams was found. Comparing PRF 100 Hz and 400 Hz data revealed a noticeable dose rate effect. Since the linac’s load differs at these frequencies, active cooling operates to maintain a steady-state temperature. As anticipated, the most significant dose rate effect was observed in the *x*-direction, which lacks servo-based beam steering.

The data exhibited sensitivity to the number of monitor units (MU) used for each exposure. As MU increased, the number of linac pulses also increased proportionally, resulting in a higher load on the linac. Effective cooling becomes crucial for the linac to operate independently of MU. At low MU, there is an expectation of larger uncertainty in the FSP measurements. However, no general pattern with MU was discerned in the data.

A dependence of the FSP on the direction of gantry rotation was observed. Specifically, a difference between CW and CCW rotation was primarily found in the *x*-direction. This effect could be attributed to gravity and the absence of servo-based beam steering in that direction, resulting in a lack of compensation for gravity’s impact on linac components, such as the bending system, waveguide, and flight tube.

The quantities ${\Delta }_{\gamma ,\text{max}}$ and ${\Delta }_{\gamma ,\pm 180}$ ($\gamma =x \text{or} y$) were used to characterise the linac’s performance and stability, respectively. As per our knowledge, adjusting the ${\Delta }_{\gamma ,\text{max}}$ value through parameter settings in the LCS software Integrity appears to be impossible. Therefore, the ${\Delta }_{\gamma ,\text{max}}$ value seems to reflect an inherent feature of the linac, relating to central components such as the waveguide and bending systems and their mechanical rigidity. Thus, ${\Delta }_{\gamma ,\text{max}}$ serves as a parameter that establishes the limits of the radiation delivery accuracy over the linac’s lifetime. On the other hand, the ${\Delta }_{\gamma ,\pm 180}$ parameter describes the stability of the FSP during beam delivery through a 360° arc. This is characterised as a hysteresis effect over time during beam delivery, indicating the FSP’s stability or deviations during gantry rotation.

FSP measurement appears to be independent of the type of panel used (i.e. AL or AP panels).A difference could arise due to varying image acquisition times mentioned in the iViewGT manual [37] which was not noticed in this study since the acquisition times for both panels were almost identical.

The main difference between the panels lies in their capabilities. The AP panel allows the acquisition of 6 MV FFF at 400 Hz, whereas the AL panel does not support this functionality. Consequently, to enable the acquisition of 6 MV FFF beams on the AL panel, the PRF was set to 100 Hz. Despite the differences in capabilities, the FSP measurement remains consistent regardless of the panel type used.

### STARCHECK phantom

The variation of the RFC measured using the STARCHECK phantom can be modelled by combining Eqs. (4)–(6). The sag of the phantom could be added as a phenomenological parameter to the BLD sag in Eq. (6). This would not change the number of fitting parameters in the model. The model describes a periodic harmonic variation of the RFC, ${\mathbf{r}}_{\text{RFC}}$, over 360° gantry rotation. The data shown in Figs. 8a–f display partly harmonic variations. Higher harmonic components occur, as well as mechanical hysteresis effects caused by gravity. The model serves as a simplified description of the RFC position during gantry rotation, characterising the major contributions.

The calculation of beam symmetry uses the calculated RFC as the reference point (zero). The symmetry was measured for 20 × 20 cm^2^ field size, and an RFC variation of < 2 mm would, in a naive estimate, result in symmetries < 2%. The symmetry follows this estimate, but a strong correlation between the RFC and symmetry does not seem to be present.

Using a 2D-array, such as the PTW STARCHECK phantom, to detect FSP by measuring RFC during gantry rotation has limitations due to the possible impact of sag in the BLD and holding rack on the results. However, assuming that the sag effects are independent of the beam, the STARCHECK phantom could still be employed to determine the relative variation compared to a reference energy, *i.e.*, 6 MV. This approach would enable the assessment of FSP variation relative to the 6 MV beam. In addition, the STARCHECK phantom could be a valuable tool for evaluating the dose rate dependency under gantry rotation compared to a reference dose rate. By leveraging its capabilities, this phantom facilitates the examination of dose rate variations during gantry rotation and provides insights into the dependency of dose rate on different beam energies.

### ArcCHECK phantom

The ArcCHECK phantom is designed to measure the accumulated dose distribution, and it cannot directly interpret the RFC or FSP as a function of the gantry angle. Instead, the accumulated dose distribution is compared to a reference distribution using the SNC software, which calculates the pass rate. These pass rates are highly sensitive to the alignment of the phantom, as shown in Fig. 10b, d. Proper alignment is crucial to ensure accurate and reliable pass rate calculations.

In Eq. (5), the parameter ${a}_{x}$ does not affect the isocentre, as shown in Eq. (7). However, it is anticipated that the ${a}_{x}$ parameter will influence the pass rate ${P}_{\text{AD}}$. This influence is attributed to its role in extending the irradiated volume with less steep dose gradients, leading to penumbra broadening.

The mechanical sag and beam steering vary with the gantry angle, resulting in a slight shift of the linac isocentre relative to the idealised isocentre represented by the TPS, as described in Eq. (7). By using values of ${c}_{x}, {a}_{y},{b}_{x}$ from Eq. (7). The values for 6 MV FFF in linac G, are found in Fig. 6d to be ${b}_{x}\cong -0.25$ mm, ${c}_{x}\cong 0.00$ mm, and ${a}_{y}\cong -0.25$ mm, while the BLD sag amplitude at isocentre distance is estimated to be $0.25$ mm for both MLC and jaws, based on the maximum range of 0.5 mm[7]. Thus, by inserting these data and values from Table 2 into Eq. (7), the isocentre shift is estimated as $\left(\Delta {X}_{\text{I}},\Delta {Y}_{\text{I}},\Delta {Z}_{\text{I}}\right)\cong \left(0.00, 0.28, -0.58\right) \text{mm}$ and $\left(\Delta {X}_{\text{I}},\Delta {Y}_{\text{I}},\Delta {Z}_{\text{I}}\right)\cong \left(0.00, 0.45, -0.41\right)$ mm for collimator angles of 0° and 90°, respectively. Additional corrections to the shifts $\left(\Delta {X}_{\text{I}},\Delta {Y}_{\text{I}},\Delta {Z}_{\text{I}}\right)$ are likely due to misalignments of the lasers and the ArcCHECK phantom with respect to the lasers.

According to Figs. 3a, 4a, c, the maximum FSP variation in the 6 MV and 6 MV FFF beams of linac G is at the same level, while that of the 18 MV beam is smaller. This may explain why the peak of ${P}_{\text{AD}}$ for the 6 MV and 6 MV FFF beams is located at the same coordinates $\left(\Delta {X}_{\text{I}},\Delta {Y}_{\text{I}},\Delta {Z}_{\text{I}}\right)\cong \left(1, 1, -1\right) \text{mm}$, while the 18 MV beam exhibits a different location $\left(\Delta {X}_{\text{I}},\Delta {Y}_{\text{I}},\Delta {Z}_{\text{I}}\right)\cong \left(1, 0, -1\right) \text{mm}$, Fig. 10c. The difference in $\Delta {X}_{\text{I}}$ might be due to misalignment of the sagittal room laser, while the difference in $\Delta {Y}_{\text{I}}$ might be a result of a misadjustment of the bending fine value of the linac for the 6 MV and 6 MV FFF energies. Furthermore, the fact that 6 MV and 6 MV FFF share the exact location for the maximum pass rates may result from the two beam energies using the same gantry lookup table [37].

By using additional information on the beams obtained with the rigid ball-bearing phantom, it was possible to establish a reasonably good agreement between the model estimated and measured differences between the plan isocentre and linac radiation isocentre.

### Linac beam steering and performance

The maximum dose rate occurs at a certain gun current. Over time, the dose rate decreases due to wear on the gun filament, resulting in a decrease in the gun filament current as a consequence. An increase to a certain level indicates the end of life for the gun filament. However, a gun servo test is conducted bi-annually according to the Elekta rate [38] to check the state of the gun filament. Occasionally, the gun has been adjusted in between maintenance periods if the dose rate has been found to be lower than the nominal value. Therefore, the shifts in FSP are likely related to bi-annual gun servo tests, along with water tank measurements where slight beam symmetry adjustment often occurs.

Based on our experience, the estimated lifetime rule for the gun filament and the magnetron is 5 years and 2 years, respectively, for linacs used on a daily basis and mainly for VMAT treatments. A replacement of essential components in the linac, such as the gun filament, magnetron, and ion chamber, might result in a shift in the FSP baseline. Therefore, changes in FSP are likely to be observed after such replacements.

Repairs on the linacs, such as changing the ionisation chamber, magnetron, or gun filament, may affect the FSP. Long-term stability was only considered for linacs C, G and H. Major repairs and adjustments that might have affected the FSP are listed in Table 4. The data listed are extracted from an internal logbook with manual entries. The FSP method described here was not used to check the FSP after the replacement or adjustments of the linacs. The impact of replacement and adjustments carried out on linacs G and H, as shown in Table 4, might explain the changes in **d,** as shown in Fig. 6c, e, respectively. Figure 6f covers the effect of ion chamber replacement on linac H.

**Table 4:** Day numbers (#) of linac repairs and adjustments of linac C, G and H that might impact the FSP. Blank fields mean no major repairs or adjustments

Linac	C	G	H
Ion chamber replacement	–	–	#867
Magnetron replacement	–	–	#167, 604
Gun filament replacement	–	#332	–
2 T adjustment 6 MV FFF	–	#151	–
BF adjustment 6 MV FFF	–	#151	–

No data of the FSP as a function of gantry angle were acquired during the commissioning of the linacs. Therefore, it is not known whether the data for the FSP accurately represent the circumstances at the time of commissioning. The measurements presented here were all obtained at least three years after commissioning. However, it was found that minor adjustments to the beam could improve the FSP relative to the collimator axis of linac A, Fig. 2b. Thus, all the investigated linacs can meet a 1 mm tolerance for FSP averaged over 360° in gantry rotation. The ${\Delta }_{x,\text{max}}$ value, Figs. 3a, 4a, c, for 6 MV FFF was found to be larger than for the 6 MV and 18 MV. The more considerable variation in FSP for the 6 MV FFF is challenging for fulfilling the tolerance at all gantry angles, Fig. 2a. In conclusion, the beam performance during gantry rotation for 6 MV FFF is found to be more critical than for 6 MV and 18 MV.

The temperature control of major components in the linac, such as the magnetron, waveguide, gun filament, and bending magnets, is deemed important for stable beam delivery. The different Elekta/Philips linacs investigated in this work are controlled by a number of different set parameters [37, 39]. Compensation for slight variations due to beam load and sag of the magnetic system, waveguide, and flight tube is achieved by the gun and dipole magnets (2R) with servos [37, 40]. Effects caused by gravity and the sag of linac components and the attached phantom are unavoidable. However, the sag of the ball-bearing phantom was previously found to be negligible [16]. The servos operate with tolerances and uncertainties and have the ability to affect the FSP in both directions. The bending fine (BF) parameter sets the position at which the electron beam hits the high Z-target material, i.e. the position of the FS in the longitudinal direction ($y$) [37]. An increase in BF results in the FS moving towards the gun while the radiation central axis at the isocentre moves in the opposite direction.

## Conclusion

Comprehensive data have been presented on the FSP of eight Elekta linacs under gantry rotation. These data were extracted from a substantial number of EPID images acquired with two ball-bearings situated at different distances from the radiation source in a specialised rigid phantom. The FSP has demonstrated stability over short durations, with consistency day-to-day. However, over more extended periods, such as months, modest fluctuations in the FSP have been observed. These changes were small and related to linac parameter adjustments and replacement or wearing of parts important for beam generation. The largest ranges in FSP were found in association with 6 MV FFF beams. Especially, one retrofitted linac with 6 MV FFF beam energy exhibited the largest CR range ${\Delta }_{x,\text{max}}<1.50$ mm. For all other energies and linacs, the CR and IN ranges remained within ${\Delta }_{x,\text{max}}<0.50$ mm and ${\Delta }_{y,\text{max}}<0.25$ mm, respectively.

In comparison to the MLC calibration uncertainties of 1 mm for all gantry angles, the FSP remained within 1 mm from the collimator axis for 6 MV and 18 MV beams. However, more considerable deviations were observed for the 6 MV FFF in the lateral direction, remaining within 2 mm over an extended three-year period.

The radiation head-mounted PTW STARCHECK phantom was found to have the ability to extract RFC data during gantry rotation. However, the sag of the BLD and the rack holding the STARCHECK phantom make it uncertain whether the RFC data can be transformed into FSP. However, the STARCHECK phantom could be used for constancy checks via comparison to a reference baseline.

In contrast, the SNC ArcCHECK phantom positioned on the couch and irradiated with 360° VMAT plans proved unsuitable for the detection of the FSP properties of the linac. The measurements rely on the phantom setup relative to the room lasers. The lasers and the phantom setup cannot be set to sub-millimetre accuracy by eye.

In this work, the FSP was analysed in a rotated coordinate system with the $z$-axis coinciding with the collimator axis. The FSP mainly describes the performance of the linac beam steering, with sag components of BLDs and gantry excluded from the FSP assessment. Surprisingly, no recent literature recommendations for tolerances associated with the FSP exist. From a clinical perspective, the FSP appears less relevant than the CAP at the isocentre, relative to a fixed room coordinate system during gantry rotation. The performance of CAP is subject to annual testing using the MLC spoke test [1], which is a CR evaluation and not applicable to in-plane assessments. It is recommended to establish tolerance levels for the CAP during gantry rotations, reflecting the contemporary dynamic usage of the linac in both directions and for jaws as well. In addition, it would be relevant to define methods for measuring CAP in the IN direction. The CAP contains the FSP and sag effects of the linac. Therefore, if a linac fails to pass the CAP tolerance criteria, it would be relevant to investigate the beam steering further in terms of FSP measurements to find out if any improvements are possible and understand the root causes of tolerance exceeding relative to the CAP.
